# The Invisible Burden: Self-Care and Stress in Dual Caregivers Working in Long-Term Care

**DOI:** 10.1093/geroni/igaf122.3669

**Published:** 2025-12-31

**Authors:** Karen Hirschman, Charlotte Weiss, Mary Naylor

**Affiliations:** University of Pennsylvania, Philadelphia, Pennsylvania, United States; University of Pennsylvania, Philadelphia, Pennsylvania, United States; University of Pennsylvania, Philadelphia, Pennsylvania, United States

## Abstract

Long-term care (LTC) employees often juggle caregiving roles at work and home, yet the effects of this dual responsibility are not well understood. This study examined the experiences of LTC employees who also provide unpaid care to family or friends, using surveys and interviews conducted as part of an employer-based support program evaluation. At baseline screening, 107 of 143 employees (75%) reported caregiving outside of work, with nearly half providing over 30 hours per week. Among survey respondents (N = 76), high levels of self-care neglect were reported, with most indicating low to very low engagement in managing, maintaining, or monitoring their own self-care. Stress levels were moderate for 60% and high for 30% of participants. Employees reported high self-care neglect and low to very low management, maintenance, or monitoring of their self-care. Stress levels were moderate for 60% of employees, with an additional 30% reporting high stress. Despite the support program, health self-care neglect worsened over time for some employees (p = 0.01), with no changes in other self-care measures or stress. Employee interview transcripts were coded using a conventional content analysis approach. The key theme identified, “Caregiving is my life, caregiving all around,” was supported by subthemes: “I take care of everything,” “Work at work, work at home,” and “Always something to do, always doing for others.” These qualitative findings illustrate the quantitative findings. Suggestions for developing employer-based support programs that align with the lived experiences of LTC employees who also care for family members or friends will be presented.

